# Characterisation of charred organic matter in micromorphological thin sections by means of Raman spectroscopy

**DOI:** 10.1007/s12520-020-01263-3

**Published:** 2021-01-06

**Authors:** Glenn Lambrecht, Caterina Rodríguez de Vera, Margarita Jambrina-Enríquez, Isabelle Crevecoeur, Jesus Gonzalez-Urquijo, Talía Lazuen, Gilliane Monnier, Goran Pajović, Gilbert Tostevin, Carolina Mallol

**Affiliations:** 1 Instituto Universitario de Bio-Orgánica Antonio González (IUBO), Universidad de La Laguna, Santa Cruz de Tenerife, Spain; 2 Departamento de Biología Animal, Edafología y Geología, Universidad de La Laguna, Santa Cruz de Tenerife, Spain; 3 Université de Bordeaux, CNRS, UMR 5199 - PACEA, Pessac, France; 4 Instituto Internacional de Investigaciones Prehistóricas de Cantabria (IIIPC), Universidad de Cantabria, Santander, Spain; 5 Department of Anthropology, University of Minnesota, Minneapolis, MN USA; 6 National Museum, Cetinje, Montenegro; 7 Departamento de Geografía e Historia, Universidad de La Laguna, Santa Cruz de Tenerife, Spain

**Keywords:** (micro-)Raman spectroscopy, Char, Tar, Charcoal, Micromorphology, Archaeology, Fire, Pyrotechnology

## Abstract

Burned or charred organic matter in anthropogenic combustion features may provide important clues about past human activities related to fire. To interpret archaeological hearths, a correct identification of the organic source material is key. In the present work, Raman spectroscopy is applied to characterise the structural properties of char produced in laboratory heating- and open-fire experiments. This reference data set is compared to analyses of three different archaeological sites with Middle Palaeolithic combustion contexts. The results show that it is possible to determine whether a charred fragment is the product of burning animal-derived matter (e.g. meat) or plant-derived matter (e.g. wood) by plotting a few Raman spectral parameters (i.e. position of G and D bands, and intensity ratios *H*_D_/*H*_G_ and *H*_V_/*H*_G_) against one another. The most effective parameters for discriminating animal- from plant-derived matter are the position of the G band and the *H*_V_/*H*_G_ intensity ratio. This method can be applied on raw sample material and on uncovered micromorphological thin sections. The latter greatly compliments micromorphology by providing information about char fragments without any clear morphological characteristics. This study is the first of its kind and may provide archaeologists with a robust new method to distinguish animal- from plant-derived char in thin sections.

**Supplementary Information:**

The online version contains supplementary material available at 10.1007/s12520-020-01263-3.

## Introduction

The identification of burned organic matter in anthropogenic combustion features is essential for interpreting archaeological hearths (Mallol et al. 2017). Charred particles in particular may provide valuable clues about fuel, food or refuse from different human activities. To interpret a hearth’s function correctly, it is crucial to be able to identify the source organic material from which those particles originated (e.g. bone, animal fat, wood, wood tar etc.).

Micromorphology has proven to be a reliable method for identifying mineral and organic components in combustion features (e.g. Nicosia and Stoops 2017). This method involves the observation of thin sections of undisturbed archaeological sediment through a petrographic microscope. As such, the spatial and stratigraphic relationship between components remains intact. Under the microscope, charred particles (hereafter referred to as ‘char’) appear opaque in transmitted light. The current practice is to classify char based on morphological criteria (Mallol et al. 2017). For example, char that is the product of wood combustion is identified by the presence of a cellular structure, reminiscent of woody tissues. This type of char is named ‘charcoal’. Char fragments that have a vesicular structure and drop-like morphology have been interpreted by Goldberg et al. (2009) as the product of burned fat or flesh. This type of char is referred to as ‘fat-derived char’. Recently, however, Huisman et al. (2019) have observed char with a very similar morphology attached to charcoal fragments, which they interpreted as solidified wood tar. This presents a problem within micromorphology: in the absence of supplementary information (e.g. presence of bones etc.), loose vesicular char fragments with drop-like morphology can either be interpreted as animal-derived matter (e.g. fat-derived char) or plant-derived matter (e.g. wood-derived tar), obviously changing the interpretation of hearth function. Also, when char fragments do not possess any clear morphological characteristics, identifying their organic source material is impossible without supplementary information. Thus, a purely micromorphological approach can in some cases not identify the origin of burned organics unequivocally.

Over the years, micromorphology has been complemented with other microscopy-based techniques, the sum of these methods often referred to as the ‘micro-contextual approach’ (Goldberg and Berna 2010). The current practice is to apply a combination of micromorphology, micro-Fourier transform infrared (FTIR) spectroscopy, and organic petrology. Micro-FTIR probes the molecular structure of matter. Within the micro-contextual approach, it is applied to discriminate between burned and unburned bones and to identify inorganic soil components (e.g. Stiner et al. 1995; Goldberg and Berna 2010; Stahlschmidt et al. 2015; Villagran et al. 2017). To our knowledge, it has not yet been applied on char even though char spectra have been reported and published in geological sciences (e.g. Guo and Bustin 1998). Organic petrology is the study of carbonaceous matter in sediments (e.g. Taylor et al. 1998). Individual components are referred to as ‘macerals’ and studied under oil immersion with a petrographic microscope in reflected-optical and fluorescent mode (Ligouis 2017). Within the micro-contextual approach, organic petrology is able to confirm whether macerals are likely to be the product of burning (i.e. char) or of humification (e.g. Taylor et al. 1998; Goldberg et al. 2009; Stahlschmidt et al. 2015; Ligouis 2017; Villagran et al. 2017). While both micro-FTIR and organic petrology provide useful information, none of these methods—to this date—have been explored to distinguish animal- from plant-derived matter in archaeological contexts.

Other (non-microscopic) techniques, such as molecular and (compound-specific) isotopic analyses of archaeological sediment have recently also been applied to detect the presence of animal- or plant-derived matter in anthropogenic combustion contexts (e.g. Buonasera et al. 2019; Jambrina-Enríquez et al. 2019). While these techniques are powerful, they cannot be directly applied on individual microscopic char fragments in thin sections. They also require relatively large amounts (a few grams) of sample material, which in practice constrains their stratigraphic resolution to a centimeter scale.

In the present work, we explore Raman spectroscopy as a complementary technique to micromorphology for the characterisation of char in combustion features. As this method is also microscopy based, it is directly applicable on individual microscopic char fragments either as raw sample material or embedded in uncovered micromorphological thin sections. Our aim is to quantify and interpret differences between Raman spectra from different char types (i.e. chars derived from meat and wood of different animal and tree species). The present study thus focusses on identifying the source organic material from which char could be derived (i.e. animal- or plant-derived matter).

## Background to Raman spectroscopy of char

Raman spectroscopy is a laser-based microscopy technique that provides information about molecular structures by measuring the vibrational energies of covalent bonds between atoms (e.g. Smith and Dent 2005). Char and charcoal are products of heating biomass in the absence of air (i.e. carbonisation) or under conditions that have a limited supply of oxygen (i.e. incomplete combustion or charring) (Braadbaart and Poole 2008). They may also be referred to as ‘pyrogenic organic matter’ (Zimmerman and Mitra 2017) or ‘black carbon’ (Goldberg 1985). Black carbon encompasses a range of products: from slightly charred, degradable biomass, to highly condensed, refractory soot (Masiello 2004). Two mechanisms of formation can be distinguished, resulting in two fundamentally different molecular structures: a first mechanism entails a recombination of small molecules (volatiles) released by pyrolysis into larger molecules and polycyclic aromatic hydrocarbons (PAH). A few PAHs form stacks of about 0.1 × 2 × 2 nm (Heidenreich et al. 1968; Schmidt and Noack 2000). These stacks form a network of spheroidal units of about 20 nm in diameter (Heidenreich et al. 1968) which precipitate on a surface, forming aggregates. This type of char is commonly referred to as ‘soot’ (Kennedy 1997). The second mechanism is lesser known and involves an in situ transformation of the solid source material into progressively larger aromatic structures until a network of similar randomly oriented stacks is formed—the difference being that these stacks do not form spheroidal units. At a microscopic scale this char mostly retains the morphology of its precursor material (Schmidt and Noack 2000; Masiello 2004). While soot consists of mostly carbon (Kennedy 1997), charcoal produced at temperatures below 700 °C may still contain substantial amounts of oxygen and hydrogen (Cao et al. 2012).

The Raman spectra of char are mainly characterised by in-plane vibration of carbon atoms in aromatic structures (sp^2^ hybridisation). This vibration produces a peak at about 1575–1600 cm^−1^. In ideal graphite crystals, it is the only vibration that is Raman active and is called the ‘G’ band—G standing for graphite. As chars consist of small stacks of PAHs oriented in all directions, they do not possess a crystal structure such as graphite. Heating chars to about 2500–3000 °C, however, will slightly increase their crystallographic order but graphite will never form (Heidenreich et al. 1968; Bernard et al. 2010). In contrast, graphitisable carbons such as antracene-based cokes will transform to graphitic carbon and graphite over the same temperature range (Bernard et al. 2010). In char and graphitic carbon, a broad band at about 1350 cm^−1^ is also present. This band is broad in chars but narrower in graphitic carbons (e.g. Beyssac et al. 2003). It is caused by a double resonance effect created by defects in the ideal graphite structure (Thomsen and Reich 2000). This band is referred to as the ‘D’ band—D standing for disorder (Tuinstra and Koenig 1970; Vidano and Fischbach 1978; Wang et al. 1990). In graphitic carbons, the positions of the D and G band, as well as the relative height of the D band compared to the height of the G band (i.e. the D/G height ratio *H*_D_/*H*_G_) depends on the amount of defects in the graphitic crystal structure (e.g. Tuinstra and Koenig 1970; Vidano and Fischbach 1978; Wang et al. 1989; Beyssac et al. 2003), the size of the crystals (Tuinstra and Koenig 1970; Wang et al. 1990), the orientation of the crystal relative to the Raman laser beam (Wang et al. 1989) and the temperature and pressure of formation (e.g. Beyssac et al. 2002, 2003). In chars, lacking crystal structure, those parameters mainly depend on temperature of formation (e.g. McDonald-Wharry et al. 2013; Deldicque et al. 2016) and degree of degradation (e.g. Goler et al. 2019). Unlike the G band, the D band position is sensitive to the laser wavelength used so care should be taken when comparing Raman spectra of char acquired with different laser wavelengths (Vidano et al. 1981; Mernagh et al. 1984; Wang et al. 1990; Russo and Ciajolo 2015).

A number of papers have recently been published that are relevant to archaeology: exploiting the relation between *H*_D_/*H*_G_ and temperature of formation, Deldicque et al. (2016) proposed a palaeothermometre for wood-based chars. They show that with increasing burning temperature, *H*_D_/*H*_G_ ratios rise in a predictable manner. Dupin et al. (2019) applied this technique on micromorphological thin sections to characterise charcoal production platforms dating from the seventeenth to the twentieth century. Although char is considered to be chemically very stable, it can significantly degrade through oxidation and microbial activity over tens to hundreds of years (e.g. Bird et al. 1999). Such degradation may impact the Raman spectra, as suggested by Goler et al. (2019) who have shown that *H*_D_/*H*_G_ ratios in carbon-based inks from ancient Egyptian manuscripts decrease with increasing age of the manuscripts.

## Materials and methods

### Samples

Samples were divided into ‘reference samples’ (Table 1) and ‘unknown samples’ (Table 2). Reference samples contained char of known origin (plant- or animal-derived) and were further subdivided into samples produced in laboratory furnace experiments and samples produced in outdoor fire experiments. Unknown samples contained both plant- and animal-derived char. In these samples, individual char fragments were interpreted to be of plant or animal origin through micromorphology (see ‘Micromorphology’). Unknown samples were further subdivided into samples produced in outdoor fire experiments and archaeological samples. The latter were selected from three different sites: Axlor—Spain (43° 07′ 17″ N, 2° 43′ 41″ W), Crvena Stijena—Montenegro (42° 46′ 44.4″' N, 18° 28′ 51.6″ E) and La Roche-à-Pierrot—France (45° 45′ 1.9224″ N, 0° 30′ 21.5968″ W), with Middle Palaeolithic combustion contexts for which hearth function and fuel sources are unknown. All samples were prepared as uncovered polished thin sections suitable for micromorphological analysis. In addition, some sample material from the furnace experiments (Table 1) was left unprepared to investigate effects of thin section preparation.

**Table 1:** Experimental reference samples used in this study. All samples were prepared as uncovered thin sections suitable for micromorphological analysis. In addition, some additional sample material from furnace experiments was left unprepared (i.e. unprocessed) to investigate effects of thin section preparation (samples marked with ^a^)

Sample	Type	Description/charring conditions	Reference
Plant-derived matter			
Pine bark 350^a^	Furnace experiment	Fragments of *Pinus canariensis* bark placed ina muffle furnace set to hold temperature at 350 °C for 1 h	This study
Pine bark 400^a^	Furnace experiment	Fragments of *Pinus canariensis* bark placed ina muffle furnace set to hold temperature at 400 °C for 1 h	This study
Pine bark 450^a^	Furnace experiment	Fragments of *Pinus canariensis* bark placed ina muffle furnace set to hold temperature at 450 °C for 1 h	This study
Pine xylem 350^a^	Furnace experiment	A block of *Pinus canariensis* xylem placedin a muffle furnace set to hold temperature at 350 °C for 1 h	This study
Pine xylem 400^a^	Furnace experiment	A block of *Pinus canariensis* xylem placed ina muffle furnace set to hold temperature at 400 °C for 1 h	This study
Pine xylem 450^a^	Furnace experiment	A block of *Pinus canariensis* xylem placed ina muffle furnace set to hold temperature at 450 °C for 1 h	This study
Celtis 350	Furnace experiment	A slice of *Celtis australis* branch placed in amuffle furnace set to hold temperature at 350 °C for 1 h	Jambrina-Enríquez et al. (2018)
Celtis 450	Furnace experiment	A slice of *Celtis australis* branch placed ina muffle furnace set to hold temperature at 450 °C for 1 h	Jambrina-Enríquez et al. (2018)
Celtis NFT-13-31	Outdoor fire	Dry *Celtis* sp. wood and leaves, burnedon natural sandy soil, open fire	Mallol et al. (2013a, b)
Pine NFT-16-1b	Outdoor fire	*Pinus nigra* wood, burned on naturalsandy soil, open fire	Mallol et al. (2013a, b)
Pine EF2	Outdoor fire	*Pinus canariensis* wood, burned on artificialsubstrate of basalt sand, open fire,combustion temperature oscillated between ± 300 and 800 °C	Buonasera et al. (2019)
Animal-derived matter			
Cow meat 350^a^	Furnace experiment	A piece of *Bos taurus* meat placed in a mufflefurnace set to hold temperature at 350 °C for 1 h	This study
Cow meat 400^a^	Furnace experiment	A piece of *Bos taurus* meat placed in a mufflefurnace set to hold temperature at 400 °C for 1 h	This study
Cow meat 450^a^	Furnace experiment	A piece of *Bos taurus* meat placed in a mufflefurnace set to hold temperature at 450 °C for 1 h	This study
Rabbit meat	Outdoor fire	Rabbit meat, burned in a simple open firefuelled by *Pinus nigra* wood	Mallol et al. (2013b)
Trout meat	Outdoor fire	Trout meat, burned in a simple open firefuelled by *Pinus nigra* wood	Mallol et al. (2013b)

**Table 2:** Investigated ‘unknown’ modern and archaeological samples. All samples were prepared as uncovered thin sections suitable for micromorphological analysis

Sample	Age (BP)	Description	Charcoal	Char	Wood char	Bone char	Reference
Modern experimental fire							
NFT-10-3-1	Modern	Pork ribs with meat and calcined horse bone, burned in asimple open fire fuelled by *Pinus nigra* wood	Yes	Yes	Yes	Yes	Mallol et al. (2013a, b)
Archaeological fire contexts							
Axlor, Spain	60–40 ka	Layer N, a silty clayey anthropogenic deposit in a rock sheltercontaining cm-thick horizontal beds of lithic artefacts, burned andunburned bone, charcoal and unidentified char fragments.	Yes	Yes	Yes	Yes	González-Urquijo et al. (2006)
Crvena Stijena, Montenegro	90–70 ka	Layer XXIV, a silty clayey anthropogenic deposit in a rock shelter containingcm-thick horizontal beds of abundant charcoal and burned bone fragments,as well as few unidentified char fragments.	Yes	Yes	Yes	No	Whallon (2017)
La Roche-à-Pierrot, France	60–35 ka	Layer EGPF, a sandy clayey anthropogenic deposit in a rock sheltercontaining cm-thick horizontal beds of lithic artefacts, burnedand unburned bone fragments, and rare charcoal and unidentifiedchar fragments.	Yes	Yes	No	No	Lévêque et al. (1993)

### Methods

#### Furnace experiments

**Fig. 1:**
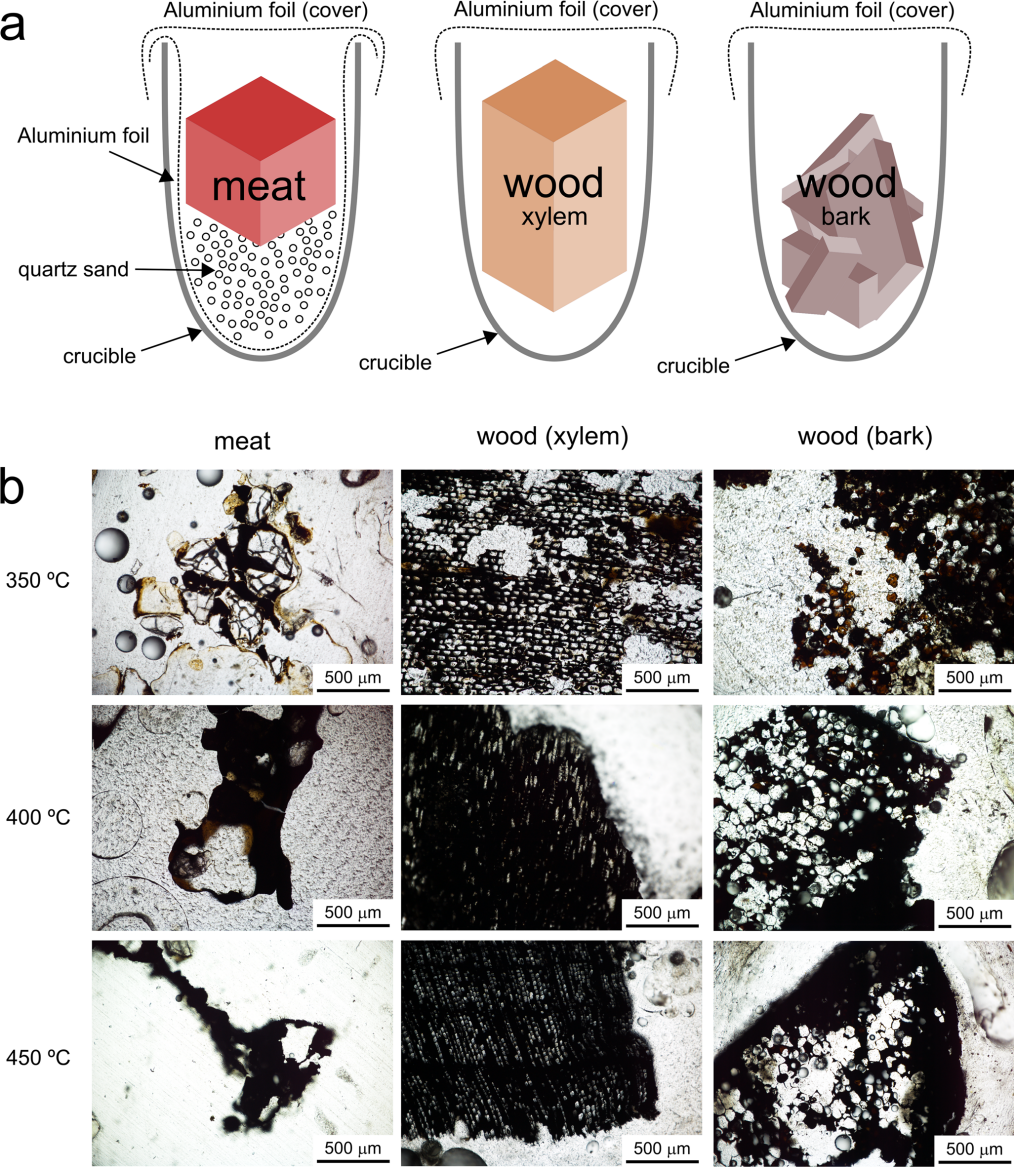
Experimental furnace samples: **a** setup of the experiment and **b** resulting thin sections

Xylem and bark samples (Table 1; Fig. 1) were prepared as follows: blocks of xylem tissue, of approximately 2 × 3 × 4 ± 0.5 cm, and weighing about 9–11 g, were cut with a handsaw from a slice of a *Pinus canariensis* tree trunk. Three such blocks were made and each placed inside a porcelain crucible, covered with aluminium foil. Three such crucibles were prepared, one for each target temperature (350, 400 and 450 °C). Fragments of bark were manually removed from the same slice of a *Pinus canariensis* tree trunk and broken into centimeter-sized fragments. Three crucibles (one for each above temperature) were filled with about 6.5 g of bark and covered with aluminium foil. Cow meat samples were prepared as follows: first, each porcelain crucible’s interior was lined with aluminium foil to allow easy extraction of char after burning. Approximately 10 g of pure quartz sand (i.e. α-quartz) was placed inside this crucible and one block of cow meat of about 16–21 g was placed on top of the sand. This way, any liquid residue produced during the experiment could be captured by the sand substrate. Then, the whole crucible was covered with aluminium foil.

Each filled crucible was weighed with a laboratory precision balance (error: ± 0.0001 g) and subsequently placed inside a muffle furnace (model Nabertherm B180). The furnace was programmed to heat to the target temperature, applying a heating rate of 18–22 °C min^−1^, and to keep that target temperature stable for 1 h. A limited amount of oxygen was present inside the furnace so combustion of wood tissue likely occurred, temporarily adding some additional heat to the sample. As the muffle furnace did not feature an active cooling mechanism, the stated temperatures should thus be considered minimum approximations of actual sample temperature. After cooling to room temperature, each filled crucible was weighed again to record sample weight loss (see Table S1 in Online Resource for weight and temperature data). A small amount of char or charcoal was removed for (raw, unprocessed) analysis. The remaining material was embedded in a two-component epoxy resin and prepared as a polished thin section without covering glass using a precision cutting machine (ATM-Brillant 2000) and grinding machine (G&N MPS-RC).

The samples ‘Celtis 350’ and ‘Celtis 450’ (Table 1) were similarly prepared in a previous study (Jambrina-Enríquez et al. 2018).

#### Outdoor fire experiments

Modern outdoor ‘NFT’ fires, ‘Rabbit meat’ and ‘Trout meat’ (Tables 1 and 2) were created as part of the Neanderthal Fire Technology Project (Mallol et al. 2013a, b). The ‘EF2’ reference fire (Table 1) was created in another study (Buonasera et al. 2019).

#### Micromorphology

**Fig. 2:**
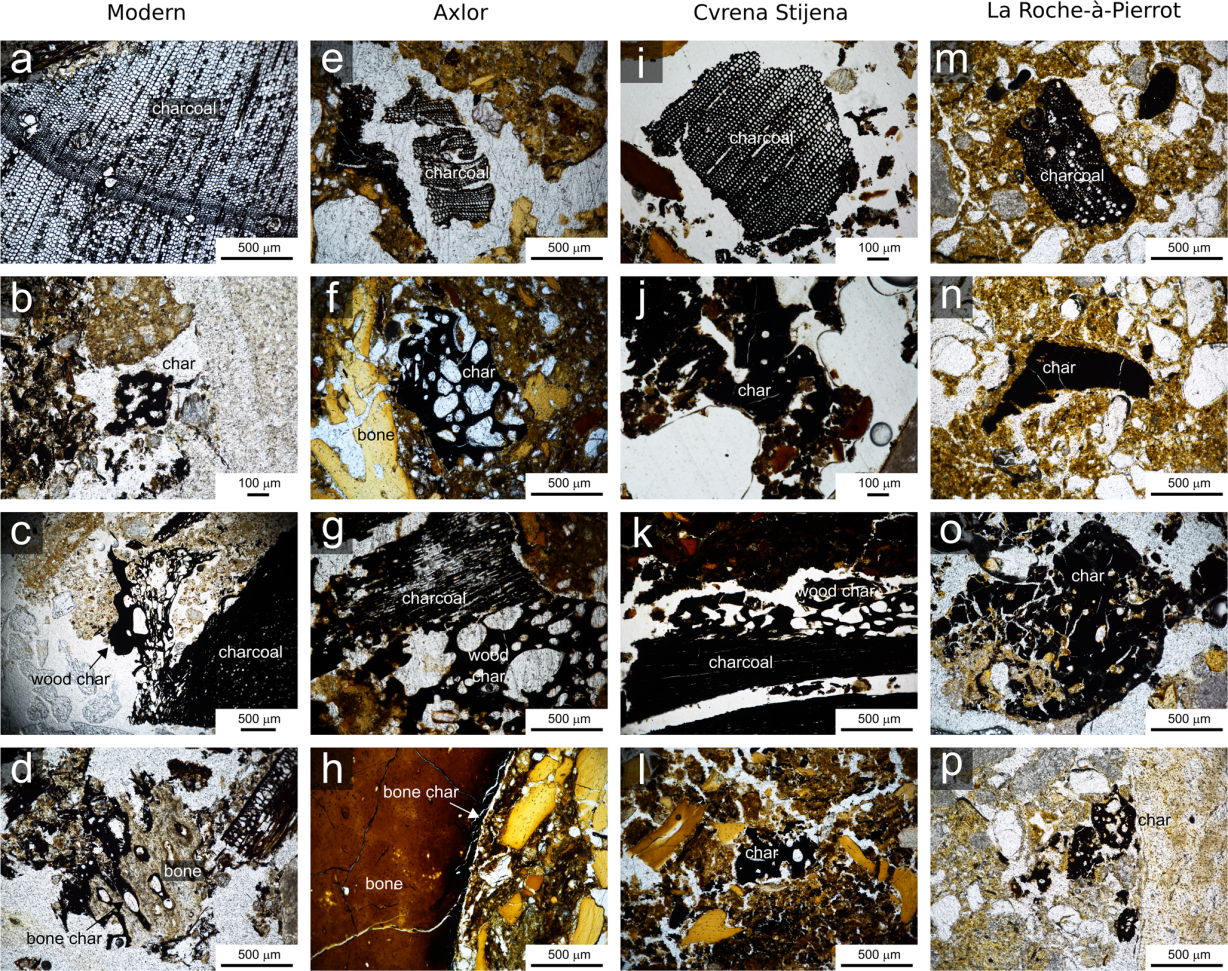
Microphotographs of unknown samples showing examples of charred fragments identified through micromorphology as charcoal, char, wood char and bone char: **a**–**d** modern (NFT) samples, **e**–**h** Axlor samples, **i**–**l** Cvrena Stijena samples and **m**–**p** La Roche-à-Pierrot samples

A Nikon AZ-100 petrographic microscope was used to characterise charred matter in unknown samples (Table 2). Criteria described in Mallol et al. (2017) were followed. Charred fragments with a clearly preserved anatomy of woody tissue were labelled ‘charcoal’. Fragments with vesicles, cracks and drop-like anatomy were labelled ‘char’. When this type of char was attached to charcoal, it was labelled ‘wood char’ and when it was attached to burned bone, it was labelled ‘bone char’ (Fig. 2). The presence or absence of these char types in samples is given in Table 2.

#### Raman spectroscopy

Raman spectra were acquired with a Renishaw InVia™ confocal Raman microscope, located at the Department of Physics and IMN of the University of La Laguna (ULL), Tenerife, Spain. The samples were excited with a 532-nm (green) laser focused on the sample surface. The Raman signal was collected in backscattered mode using a 50x objective. Sampled volume was a few cubic micrometres, and power on sample was kept low at about 0.07–0.12 mW to avoid sample degradation. The diffraction grating was 1800 l/mm, which produced spectral resolutions of 1.73 cm^−1^ per pixel. An initial set of 57 spectra was recorded from 200 to 4000 cm^−1^, and a later set of 108 spectra was recorded from 800 to 2300 cm^−1^ to cut back on overall measurement time. Acquisition time was 3 × 10 s for all spectra. For the reference samples, five measurements were made on each sample. For the unknown samples, for each sample location (i.e. modern, Axlor, Crvena Stijena and La Roche-à-Pierrot), up to five char fragments of each category (i.e. charcoal, char, wood char and bone char) were chosen, if able. Each char fragment was then measured twice.

**Fig. 3:**
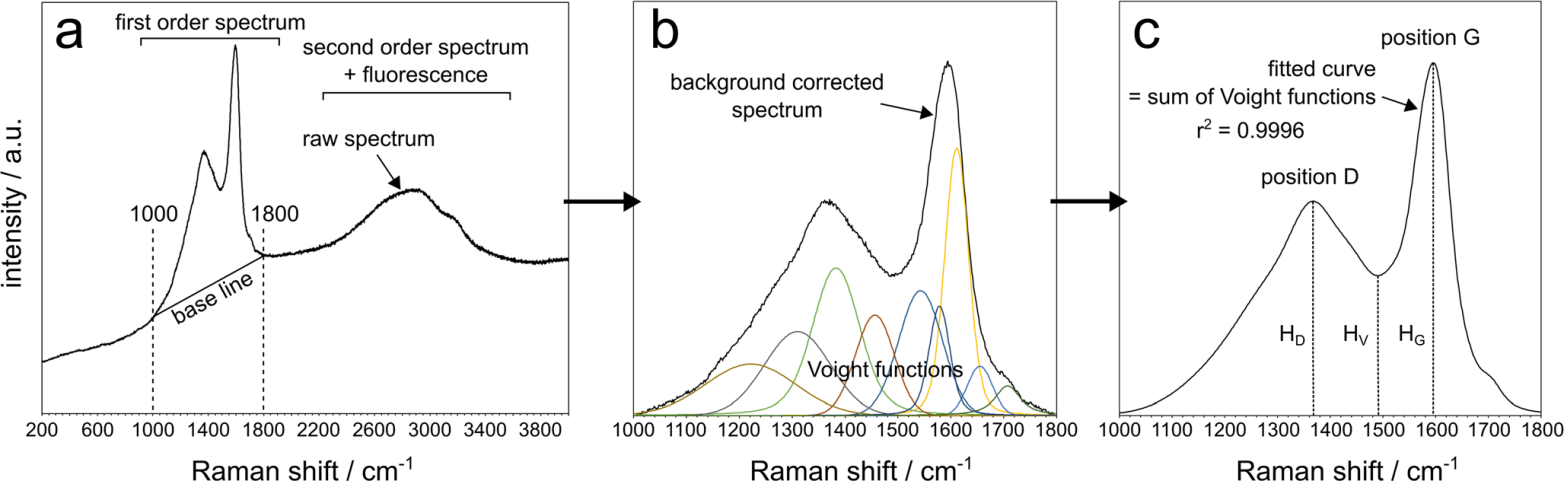
Raman spectral processing and extraction of parameters: **a** Acquisition of the raw Raman spectrum showing the first- and second-order spectrum of char. Only the first-order spectrum is considered. A background baseline is chosen between 1000 and 1800 cm^−1^. **b** Background-corrected spectrum, fitted with an arbitrary number of Voight functions. **c** Measurement of Raman parameters on fitted curve

Spectra were processed using the program PeakFit^T^™ v4.06 by AISN Software Inc. The procedure to extract spectral parameters was similar to the method described in Deldicque et al. (2016) and is shown in Fig. 3. After acquiring a raw spectrum, a section between 1000 and 1800 cm^−1^ was selected and a straight base line was drawn between the end points (Fig. 3a). After subtraction of the base line, the background-corrected spectra were fitted with an arbitrary amount (between 6 and 11) of Voight functions (Fig. 3b). The purpose of this fitting procedure was to eliminate any subjective choice of spectral parameters. Voight functions were chosen as they fitted the background-corrected spectra best (compared to Gaussian or Lorentzian functions). The *r*^2^ value, calculated by PeakFit™, was used to evaluate the quality of the fit. For all data, *r*^2^ value was above 0.995, and for the majority of data, it was above 0.999. After successful fitting, five parameters were extracted from the fitted curve (Fig. 3c): the positions of the D and G band (in cm^−1^) were defined as the highest points on the fitted curve between 1300 and 1400 cm^−1^ and 1550 and 1650 cm^−1^, respectively. The heights of these points (in arbitrary units) were recorded as *H*_D_ and *H*_G_, respectively. The height of the lowest point between D and G, *H*_V_ (‘V’ standing for valley) was recorded as well. From these heights, the following two ratios were calculated: *H*_D_/*H*_G_ and *H*_V_/*H*_G_.

Analyses that yielded a peak at about 1000 cm^−1^ were discarded as this peak is present in epoxy resin and indicates an overlap between the Raman spectrum of char and epoxy resin in the thin section. Such an overlap slightly increases the height of the G band of char due to the presence of another epoxy peak at about 1600 cm^−1^, thus producing values not representative for char.

## Results

### Reference samples

**Fig. 4:**
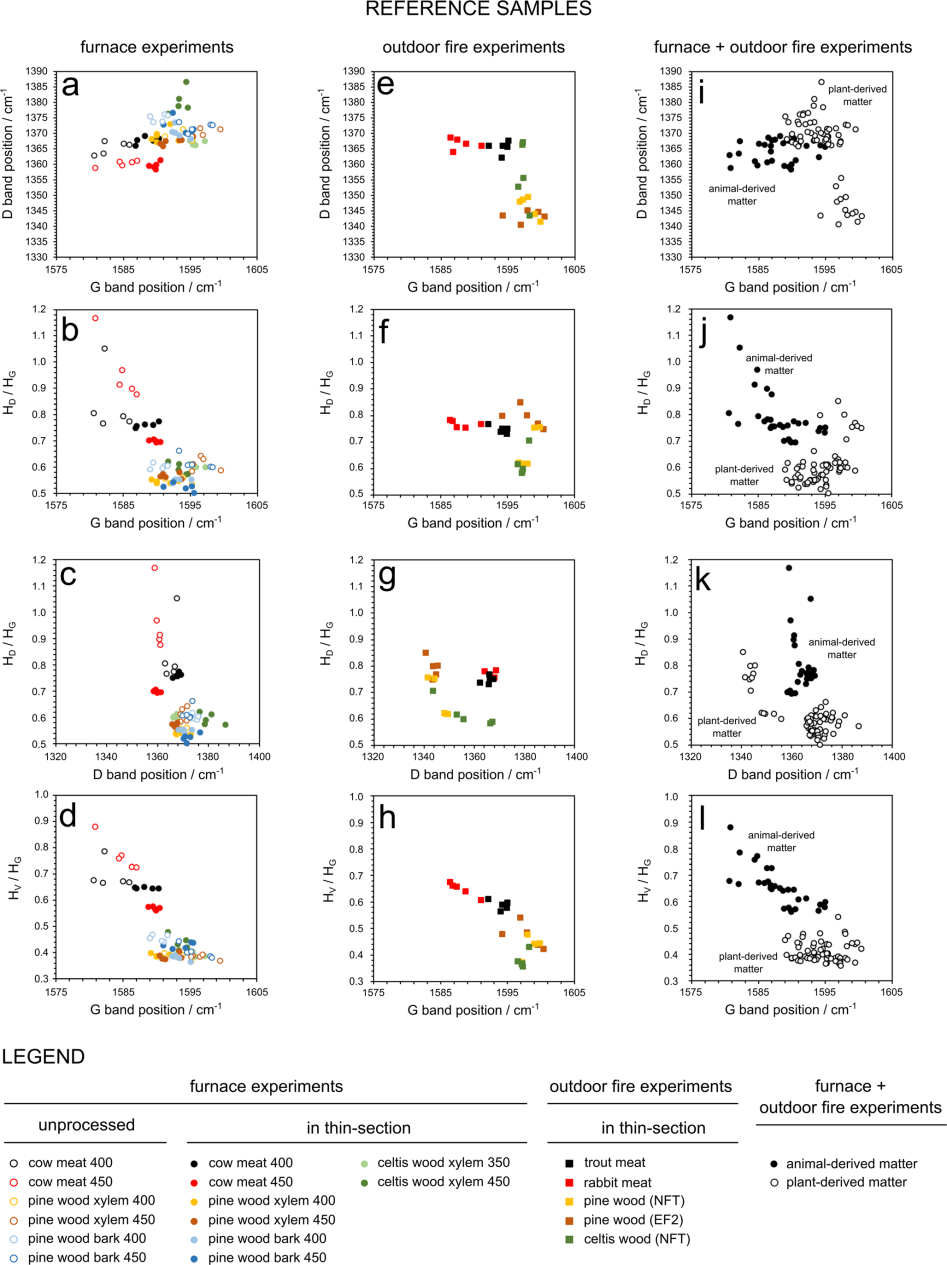
Results of reference samples: **a**–**d** samples from laboratory-heating furnace experiments, **e**–**h** samples from outdoor open-fire experiments, and **i**–**l** sum of all resulting in a full reference data set

Raman spectral parameters for the reference furnace samples are presented in Fig. 4a–d. The 350 °C samples exhibited too much fluorescence and did not show Raman bands*.* Plant-derived charcoals had G bands between 1588 and 1600 cm^−1^, a large spread in D positions, from 1355 to 1390 cm^−1^, *H*_D_/*H*_G_ ratios between 0.5 and 0.6 and *H*_V_/*H*_G_ ratios between 0.3 and 0.5. Compared to this, meat-derived chars showed lower G bands, between 1580 and 1591 cm^−1^, a smaller spread in D positions, from 1355 to 1370 cm^−1^, larger *H*_D_/*H*_G_ ratios between 0.65 and 1.2 and larger *H*_V_/*H*_G_ ratios between 0.5 and 0.9. Both *H*_D_/*H*_G_ and *H*_V_/*H*_G_ ratios allowed complete separation of plant- and meat-derived char (Fig. 4b–d).

When comparing raw unprocessed samples with polished thin sections, the following trends could be observed: plant-derived charcoals had higher G band positions when unprocessed, while animal-derived chars showed lower G bands (Fig. 4a, b). *H*_D_/*H*_G_ ratios were higher for both types when unprocessed but animal-derived samples seemed more affected by this (Fig. 4b, c). *H*_V_/*H*_G_ ratios were higher in unprocessed animal-derived char but remained the same for plant-derived charcoal (Fig. 4d).

In contrast to the furnace samples, the samples produced in outdoor fire experiments (Fig. 4e–h) had no constraint on temperature. As the Raman spectra of char are sensitive to burning temperature (e.g. McDonald-Wharry et al. 2013; Deldicque et al. 2016), a larger variety in spectral values is expected, compared to the furnace experiment samples. Similar to the furnace samples, plant-derived charcoals in outdoor fires had G bands between 1593 and 1601 cm^−1^ and a large spread in D positions but with lower values, from 1340 to 1370 cm^−1^. *H*_D_/*H*_G_ ratios were higher, between 0.55 and 0.85, and *H*_V_/*H*_G_ ratios had a larger range, from 0.3 to 0.6. Meat-derived char showed higher values for G bands, compared to their laboratory counterparts, between 1585 and 1596 cm^−1^ and a similar spread in D values between 1360 and 1370 cm^−1^. *H*_D_/*H*_G_ and *H*_V_/*H*_G_ ratios were similar to the processed samples, ranging from 0.7 to 0.8 and 0.5 to 0.7, respectively.

Combining the data of the furnace and outdoor fire experiments resulted in a reference data set for animal- and plant-derived char (Fig. 4i–l). This data set was then used to evaluate the results of the unknown samples below.

### Unknown samples

**Fig. 5:**
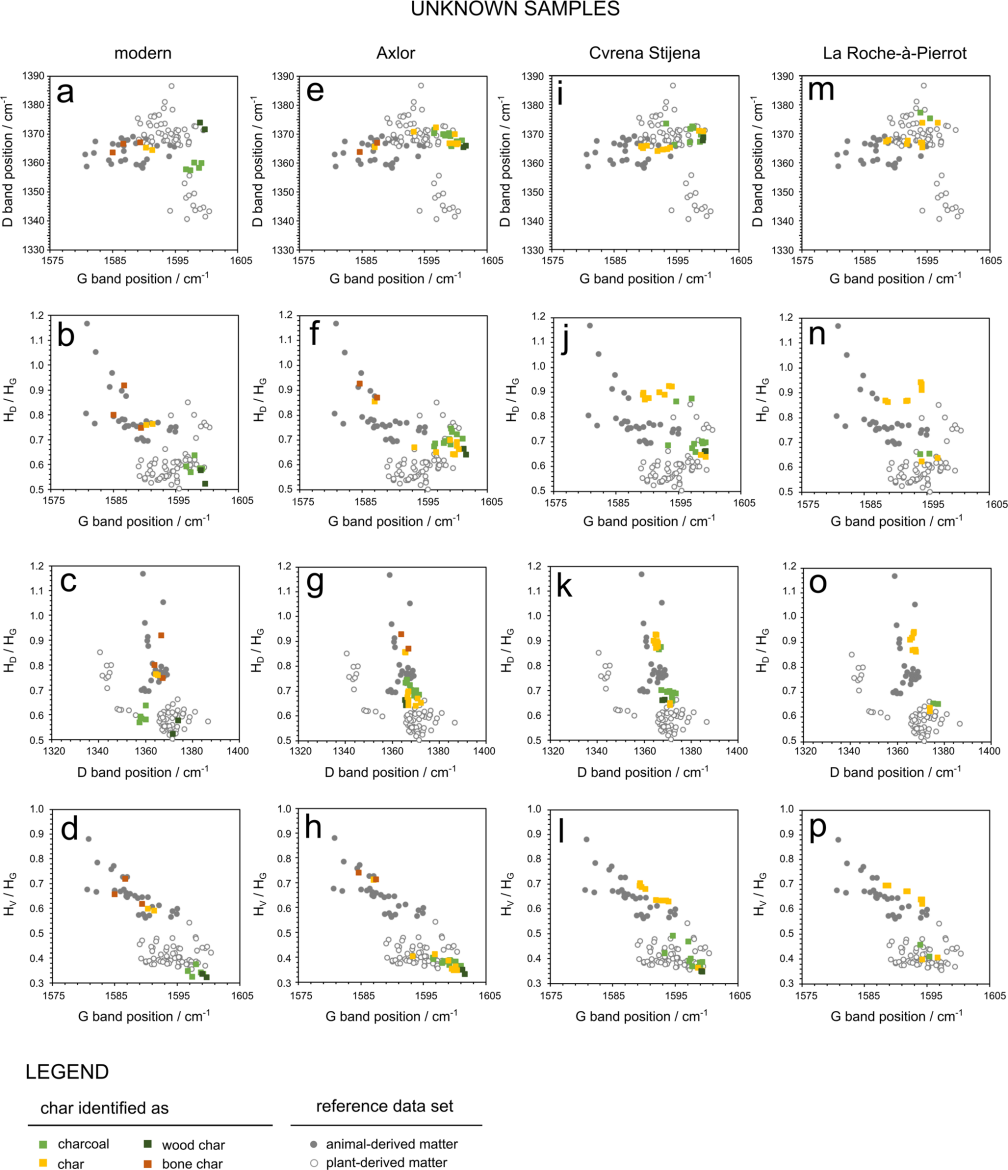
Results of unknown samples: **a**–**d** modern (NFT) samples, **e**–**h** Axlor samples, **i**–**l** Cvrena Stijena samples and **m**–**p** La Roche-à-Pierrot samples

Figure 2 shows a selection of microphotographs of charred fragments in the unknown samples (Table 2). Raman spectral parameters from these and similar charred fragments were plotted on top of the reference data set in Fig. 5.

In the modern fire samples, charcoal and wood char plot in the plant-derived matter region, while char and bone char plot in the animal-derived matter region (Fig. 5a–d). Wood char and charcoal have similar values for the G position but wood char has higher values for the D position (Fig. 5a).

Unlike modern samples, samples from archaeological fires will have undergone some degree of diagenesis, which could cause degradation of char and have an influence on Raman spectral parameters (see ‘Background to Raman spectroscopy of char’). It is thus expected that Raman spectral parameters of char in archaeological samples (Fig. 5e–p) will be somewhat different from char in modern fires. In general, charcoal and wood char plotted in the plant-derived matter region, while bone char plotted in the animal-derived matter region. Char either plotted in the animal-derived or plant-derived region (Fig. 5e–p). Compared to modern samples, charcoal from archaeological fires tended to have higher D values, similar to the values for wood char. All charred fragments in archaeological samples also seemed to have higher *H*_D_/*H*_G_ ratios, compared to modern samples (e.g. Fig. 5g, k).

### Other observations

During the impregnation step of thin-section preparation, air bubbles appeared in the two-component epoxy of the immersed plant-derived furnace samples (Table 1; Fig. 1) and the affected samples felt warm to the touch. This suggests an exothermic reaction took place between the epoxy and sample material. This phenomenon was most evident in the pine bark samples.

In most charcoal spectra, a weak band at about 1700 cm^−1^ was observed (both in modern and archaeological samples). A band at this position has been attributed to the presence of carbonyl (C=O) groups in lignin or acids (e.g. Francioso et al. 2011). Another weak band at about 2215–2225 cm^−1^, which could represent nitrile (N≡N) groups (e.g. Socrates 2001) has been observed in some of the animal-derived spectra: in unprocessed cow meat furnace samples (Table 1) and in modern bone char (Table 2).

## Discussion

### General discussion

Within the sample pool of this study, animal-derived matter (e.g. charred meat, bone char) could be easily distinguished from plant-derived matter (e.g. charcoal, wood char), using a few Raman parameters. Plotting the G band position against the *H*_V_/*H*_G_ intensity ratio particularly allowed complete separation of these groups (Figs. 4 and 5). For the present method to be applicable universally to archaeological sites, a few considerations must be made. As mentioned in ‘Background to Raman spectroscopy of char’, the Raman spectrum of char is sensitive to the temperature of formation and degree of degradation (which for archaeological sediment samples relates to post-depositional processes). Furthermore, as the samples consisted of polished thin sections, possible effects of sample preparation must be taken into account.

In micromorphological thin sections, the molecular structure of its components could be altered by mechanical polishing during sample preparation. It is well known that polishing graphitic carbon changes its Raman spectrum: Polishing induces more defects in its crystal structure and increases the height of the D band relatively to the height of the G band (Vidano and Fischbach 1978; Wang et al. 1989; Beyssac et al. 2003). The influence of polishing on the structure of chars, lacking a crystal structure, is lesser known. Considering the results of the furnace experiments in the present study (Fig. 4a–d), when comparing the raw sample material with their polished equivalents it can be observed that thin-section manufacturing has decreased *H*_D_/*H*_G_ rather than increasing it, for both animal-derived char and plant-derived charcoal (Fig. 4b). Nevertheless, there is a consistent bi-modal distribution in Raman parameters between animal- and plant-derived matter in all unknown samples (Fig. 5). If polishing had a major influence on the structure of char, we would expect the different char types identified in the unknown samples to have a more similar structure. It can thus be concluded that polishing did not cause the observed differences. This seems logical considering that char and charcoal are already poorly organised carbonaceous materials and mechanical polishing may not decrease their crystallographic order any further (Beyssac et al. 2003). Thin-section manufacturing also entails embedding the sample in resin. The position of the G band in the furnace experimental samples did change with thin-section manufacturing: in animal-derived char, the G band shifted to higher wavenumbers, while in plant-derived charcoal, it shifted to lower wavenumbers (Fig. 4a, b and d). At this moment, it is not clear what caused this shift, neither what caused the drop in *H*_D_/*H*_G_. One possibility is that embedding the raw material in resin altered the Raman spectra of our sample in some way. During preparation of the furnace samples, air bubbles were observed forming inside the epoxy during impregnation. This was most evident in the plant-derived samples. Perhaps, in those samples, pyrogenic oxygen-enriched compounds inside the sample material reacted with one of the components of the two-component resin, promoting the curation of epoxy. The presence of oxygen-rich compounds is indirectly supported by the observation that plant-derived charcoal spectra regularly show a weak band at 1715 cm^−1^, attributed to carbonyl (C=O) bonds (e.g. Francioso et al. 2011). In conclusion, thin-section manufacturing does indeed alter the Raman spectra of charred material but not in a way predicted by current understanding of disordered carbon (e.g. Beyssac et al. 2003). In any way, these changes do not appear to be large enough to make the data points of animal- and plant-derived matter overlap (Fig. 4).

The Raman spectrum of graphitic carbon is influenced by its orientation with respect to the laser beam: The relative height of the D band is at its highest point when the laser beam makes a 90° angle with the c-axis of the crystal structure, but the positions of D and G do not change (Wang et al. 1989). Char consists of stacks of PAHs oriented in every direction and no larger than a few nanometre (Heidenreich et al. 1968; Schmidt and Noack 2000). As the Raman laser excites a volume of a few cubic micrometres, it encompasses a large number of such stacks in a single measurement, and it can thus be assumed that any influence of orientation is cancelled out.

It is well known that *H*_D_/*H*_G_ in carbonaceous materials is determined by the temperature of formation (e.g. Beyssac et al. 2003). For wood-derived charcoal, Deldicque et al. (2016) have shown that *H*_D_/*H*_G_ increases in pinewood chars with burning temperature. In their experiments, the position of the D band decreases with increasing temperature up to 800 °C, and then increases above 800 °C. McDonald-Wharry et al. (2013) observe similar trends for *Pinus* wood and *Phormium* leaves: an increase in *H*_D_/*H*_G_ up to 900 °C, a decrease in D band position and an increase in G band position with increasing temperature, as well as a drop in *H*_V_/*H*_G_. Smith et al. (2016) observe similar trends in cellulose-derived chars. In the present study, furnace reference samples were constrained to a low temperature (450 °C and below) but open fires were exposed to variable (and locally higher) temperature (Table 1; Mallol et al. 2013a; Buonasera et al. 2019). When comparing the charcoal of the furnace experiments (Fig. 4a–d) with the charcoal of the open fires (Fig. 4e–h), it is indeed observed that the latter have variable *H*_D_/*H*_G_ ratios, similar and larger than the furnace experiments. A similar trend can be observed for the position of the D band (decreasing) and G band (increasing). The same trends apply to charcoal in modern unknown fires (Fig. 5a–d). We can thus attribute this distribution of data points to burning temperature. For animal-derived char, no substantial changes in Raman parameters were observed between experimental furnace and modern samples (Figs. 4 and 5a–d). In summary, even though temperature of formation creates variability in the Raman data, it does not make animal- and plant-derived matter overlap. Note that heat generated by an exothermic reaction occurring during epoxy curation (as observed in plant-derived furnace samples, see ‘Other observations’) would by itself not be sufficiently high to alter the molecular structure and Raman spectrum of char.

A final consideration is the effect of char degradation through post-depositional processes in archaeological sites. Oxidation and microbial activity are processes able to break down char (Schmidt and Noack 2000). In well-aerated tropical soil environments, charcoal can be significantly degraded in only ten to hundreds of years (Bird et al. 1999). Masiello (2004) suggests that smaller particles may be more easily oxidised, leading to a change in size-fraction over time. Inoue et al. (2017) assume that charcoal fragments from grasslands, produced under higher temperatures are more chemically stable but less physically stable, resulting in their lower rate of residue as fragments in soils as well. Francioso et al. (2011) show that the smallest charcoal size-fractions from a recently burned *Pinus pinea* forest have the highest *H*_D_/*H*_G_ ratio. In the present study, *H*_D_/*H*_G_ values of archaeological char and charcoal are higher compared to modern samples (Fig. 5e–p). Based on the above studies, this would thus correspond to a higher temperature of formation, which would make these fragments chemically more stable (thus likely to be preserved), but physically less stable (thus likely to turn into smaller fragments over time). As smaller charcoal size fractions are also correlated with higher *H*_D_/*H*_G_ ratio, we can thus hypothesise that this explains the trends observed. We suggest that compared to modern samples, larger char fragments from archaeological sites have been broken down over time, increasing *H*_D_/*H*_G_. However, this effect does not obscure the difference between animal- or plant-derived samples.

In conclusion, the above paragraphs establish that the bi-modal distribution that allows animal- and plant-derived matter to be distinguished by Raman spectroscopy is not caused by sample preparation, sample setup, burning temperature or post-depositional processes. The differences can thus be considered as representative of a true difference in molecular structure between animal- and plant-derived charred matter.

### Implications for micromorphology and archaeology

How does Raman spectroscopy compare to traditional micromorphology for classifying char fragments as ‘animal derived’ and ‘plant derived’? All fragments that were unambiguously identified through micromorphology as being of animal- or plant-derived origin (i.e. those fragments labelled as ‘bone char’, ‘charcoal’ and ‘wood char’) plotted in the corresponding regions of the Raman reference data set (Fig. 5). This supports the validity of this data set. Only fragments labelled as ‘char’ plotted either in the animal- or plant-derived region. This is not surprising as ‘char’ was defined as loose fragments with a vesicular drop-like morphology, cracks and no clear cellular structure (see ‘Micromorphology’). As stated in the ‘Introduction’, this morphology type has previously been interpreted as either animal- (fat-)derived char (Goldberg et al. 2009) or plant-derived tar (Huisman et al. 2019). Herein lies an explanation for the results; char plotting in the animal-derived region, being derived from fat or meat, and char plotting in the plant-derived region, derived from tar or from other anatomical parts of vegetative matter. Raman spectroscopy thus appears to be a great complement to micromorphology in archaeological contexts where ambiguity exists concerning the origin and nature of char fragments.

Finally, the results of the present study may be significant for the three archaeological sites considered in this study (Axlor, Cvrena Stijena, La Roche-à-Pierrot; Table 2; Figs. 2 and 5). These sites are currently under geo-archaeological microstratigraphic investigation and in each case, characterising the microscopic char components is key. The sampled contexts represent Middle Palaeolithic occupation surfaces with remains of anthropogenic combustion, as evidenced by the presence of burned bone and charcoal fragments. The results of this study suggest that, despite them being contexts with abundant burned bone, the three sites preserve microscopic char fragments derived from burning of both animal and plant matter. This information will gain behavioural meaning when contextualised in forthcoming geo-archaeological analyses. For now, the data raises a few issues that motivate further investigation. The Axlor and Crvena Stijena deposits, which both contain abundant charcoal, burned and unburned bone fragments, preserve common microscopic wood char fragments resembling tar—as described by Huisman et al. (2019). Further multi-proxy micro-contextual investigation of the combustion contexts is necessary to approach hearth function and elucidate whether this alleged tar is a natural fuel by-product or may be artificially produced. Neanderthal artificial tar production has previously been proposed in several publications (Boëda et al. 2008; Degano et al. 2019; Niekus et al. 2019). However, Raman spectra of wood tars typically show strong fluorescence or an absence of Raman bands (e.g. Tóth et al. 2018) and our wood char spectra clearly showed G and D bands. Further research is thus required to better understand the possible origins of these fragments. Fragments identified as char are predominantly plant derived in Axlor, while they are predominantly animal derived in Crvena Stijena (Fig. 5). In the latter site, Jambrina-Enríquez et al. (2019) have recently suggested the presence of both animal-fat and wood-oil combustion residues as evidenced by compound-specific carbon isotope analyses of lipids. In their study, they observe an isotopic signature attributed to animal-fat residues in stratigraphic layer BL8, while a signature in between animal-fat and wood-oil residues was detected in layers BL10 and BL11. Our corresponding Raman data (Fig. 5i–l) indeed show the presence of three microscopic animal-derived char fragments and one plant-derived charcoal fragment in BL8 and two plant-derived fragments (charcoal and wood char) in BL11. Further measurements in both stratigraphic layers may strengthen the correspondence between lipid biomarker and Raman analyses. Note also that recent compound-specific carbon isotope data suggests a strong plant-oil signature at Axlor (Jambrina-Enríquez, personal communication). In La Roche-à-Pierrot, the archaeological context investigated here is from Layer EGPF, a deposit rich in burned and unburned bone for which the use of bone as fuel has been previously proposed (Morin 2010). Accordingly, the microscopic char samples from this context are predominantly animal derived (Fig. 5m–p). Only one single fragment labelled char (Fig. 2n) was found to be plant derived. This fragment was found in a thin section that also contained a rare piece of charcoal (Fig. 2m), suggesting it could have broken off of this or another piece. In general, the results seem to support the hypothesis of bones used as fuel (Morin 2010). A more detailed and rigorous Raman study for each of these three archaeological sites provides an exciting prospect for future research.

## Conclusions

In a series of laboratory-furnace heating and outdoor open-fire burning experiments, a distinct difference was observed between Raman spectra of charred meat and charred wood. This difference was also observed in micromorphological thin sections of three archaeological combustion contexts, dating to the Middle Palaeolithic. These results lead us to conclude that:

- Raman spectroscopy is a reliable technique to distinguish animal- from plant-derived charred matter. The distinction can be made most effectively by plotting the position of the G band and the *H*_V_/*H*_G_ intensity ratio against one another.
- The method is applicable on samples of charred matter embedded in micromorphological/petrological thin sections and across a broad range of burning temperatures.
- For the three Middle Palaeolithic archaeological contexts tested, post-depositional processes were not a significant factor.

This study supports the high potential of the multi-proxy, micro-contextual approach in archaeological science and introduces a new high-resolution technique, (micro-)Raman spectroscopy, for the investigation of archaeological combustion contexts. The method presented in this study may solve some of the ambiguities that currently exist in micromorphology, namely the interpretation of some vesicular and amorphous char fragments. It may also help in interpreting charred fragments of unknown origin, without any clear morphological characteristics. As this method is directly applicable on the surface of uncovered thin sections and consists of a single-point measurement, it allows for an in situ approach, preserving the broader stratigraphic context.

## Supplementary Information

ESM 1 (PDF 293 kb)

## Data Availability

Not applicable
