# Supplementary material for: Characterisation of charred organic matter in micromorphological thin sections by means of Raman spectroscopy
Source: Archaeol Anthropol Sci. 2021 Jan 6;13(1):13. doi: 10.1007/s12520-020-01263-3 (PMC7788033; doi:10.1007/s12520-020-01263-3)
Supplement: Supplementary file 1 — (PDF 293 kb) [file 12520_2020_1263_MOESM1_ESM.pdf]

Supplementary material for

Characterisation of charred organic matter in micromorphological thin sections by means of Raman spectroscopy

by

Glenn Lambrecht[a], Caterina Rodríguez de Vera[a], Margarita Jambrina-Enríquez[a,b], Isabelle Crevecoeur[c], Jesus Gonzalez-Urquijo[d], Talía Lazuen[c], Gilliane Monnier[e], Goran Pajović[f], Gilbert Tostevin[e], Carolina Mallol[a,g]

[a] *Instituto Universitario de Bio-Orgánica Antonio González (IUBO), Universidad de La Laguna, Santa Cruz de Tenerife, Spain*

[b] *Departamento de Biología Animal, Edafología y Geología, Universidad de La Laguna, Santa Cruz de Tenerife, Spain*

[c] *Université de Bordeaux, CNRS, UMR 5199 - PACEA, Pessac, France.*

[d] *Instituto Internacional de Investigaciones Prehistóricas de Cantabria (IIIPC), Universidad de Cantabria, Santander, Spain*

[e] *Department of Anthropology, University of Minnesota, Minneapolis, MN, USA*

[f] *National Museum, Cetinje, Montenegro*

[g] *Departamento de Geografía e Historia, Universidad de La Laguna, Santa Cruz de Tenerife, Spain*

Corresponding author: Glenn Lambrecht (glambrec@ull.edu.es)

Table S1. Temperature and weight data for experimental furnace samples (Table 1).

Sample	Temperature			Mass			
	starting *T* / °C	ending *T* / °C	Ramp / °C/min	sample / g	sample + crucible before / g	sample + crucible after / g	mass loss / g
Pine bark 350	86	350	20.3	6.4837	51.2085	48.0341	-3.1744
Pine bark 400	55	400	20.3	6.5981	50.7604	47.2224	-3.538
Pine bark 450	23	450	19.4	6.5950	46.0861	42.1098	-3.9763
Pine xylem 350	86	350	20.3	10.4250	53.8854	46.9883	-6.8971
Pine xylem 400	55	400	20.3	9.4688	43.0029	36.1839	-6.819
Pine xylem 450	23	450	19.4	9.3421	50.1888	43.0916	-7.0972
Cow Meat 350	90	350	18.6	21.6503	73.9296	55.6077	-18.3219
Cow Meat 400	80	400	21.3	16.3249	66.9045	52.098	-14.8065
Cow Meat 450	20	450	18.3	18.2336	72.5094	55.4917	-17.0177
